# Building Innovative Teams: Exploring the Positive Contribute of Emotions Expression and Affective Commitment

**DOI:** 10.3389/fpsyg.2021.732171

**Published:** 2021-11-03

**Authors:** Rita Damasceno, Isabel Dórdio Dimas, Paulo Renato Lourenço, Teresa Rebelo, Marta Pereira Alves

**Affiliations:** ^1^Faculty of Psychology and Educational Sciences, University of Coimbra, Coimbra, Portugal; ^2^Centre for Business and Economics Research (CeBER), Faculty of Economics, University of Coimbra, Coimbra, Portugal; ^3^Research Center in Business Sciences (NECE-UBI), University of Beira Interior, Covilhã, Portugal

**Keywords:** work teams, innovation, emotion, emotional carrying capacity, affective commitment

## Abstract

The current challenging organizational context demands that organizations adapt quickly and continuously in order to survive and maintain their competitive advantage. Considering this need, one of the responses given by companies has been the valorization of work teams and their capacity for innovation, as well as fostering positive skills and emergent states in employees, such as emotional carrying capacity and affective commitment, respectively. The aim of this research is thus to study the relationship between emotional carrying capacity and group innovation, considering affective commitment as the mediating variable. To test these relationships, an empirical cross-sectional study was conducted including 138 Portuguese work teams belonging to different sectors of activity, composed of 625 members and their respective leaders. The results were analyzed through structural equation modeling (SEM) and showed positive relationships between emotional carrying capacity and affective commitment, as well as between affective commitment and group innovation. In addition, the mediating role of affective commitment in the relationship between emotional carrying capacity and group innovation was also supported. Therefore, the results suggest that a work context in which members openly express their emotions contributes to reinforcing their affective attachment to the group, making them feel more involved and available to test and implement new ideas and procedures. The findings reinforce the benefits of promoting the expression of emotions and the development of healthy bonds between team members.

## Introduction

High customer demands and increased intensity of competition have led to greater complexity and rigor in organizational activities (Zheng et al., 2010). To survive in this dynamic environment, organizations must identify and manage obstacles and adapt quickly, and innovation is a key process (West and Anderson, 1996b; Popa et al., 2017). The aim of this study is to contribute to understanding how team innovation can be promoted. To accomplish this objective, the relationship between the emotional carrying capacity (ECC) in teams and innovation will be analyzed, as well as the mediating role of affective commitment regarding the team on this relationship.

Innovation can be conceptualized as the intentional introduction and application, within a group or organization, of ideas, processes, products, or procedures that are new to that unit and which are intended to benefit the individual, the group, the organization, or society at large (West and Farr, 1990). More specifically, group innovation processes seem to be a powerful strategy for enhancing an organization’s ability to adapt to change and react to competitors (Richardson and West, 2009).

In this sense, increased competitiveness and the need to take advantage of development opportunities require the coordination and application of the capabilities of all the members of the organization (Zheng et al., 2010), which led to the adoption of changed-oriented and team-based organizational designs (Rico et al., 2011). Indeed, teams have become the basic unit of operation in most companies (Zheng et al., 2010; Lourenço and Dimas, 2011; Rico et al., 2011), and teamwork is even pointed to as being the most effective way to deal with complex tasks and problems and with new challenges (Lourenço et al., 2014).

A work group/team^1^ can be defined as a set of individuals who interact frequently, are interdependent in their tasks, share responsibility for results, and identify themselves and are identified as a social entity, embedded in a broader social system (Cohen and Bailey, 1997; Lourenço et al., 2014). Thus, as a complex, dynamic and non-enclosed social entity, the group develops from the relationships among its members and between them and their surroundings. Accordingly, the behavior of the group is the result of the relationships established among its members and between them and the organization as a whole (Lourenço and Dimas, 2011).

In line with the sociotechnical approach, groups can be considered as being constituted by two interdependent subsystems, the affective and the task subsystems (Fox, 1995), with the effectiveness of the group depending on its ability to balance both subsystems. By doing so, the group will be more consistent, and will have a greater ability to respond to the demands of the environment (Richardson and West, 2009). Nonetheless, team research has focused mainly the task dimensions of team dynamics and, although the body of research on the social and affective processes and states that may affect team functioning has developed significantly over the past three decades, there is still room for further exploration (Barsade and Knight, 2015; van Kleef, 2016). Regarding team innovation specifically, greater attention has been paid to the cognitive processes that influence it, while the knowledge regarding the impact of affective variables on innovation remains relatively underdeveloped (Hülsheger et al., 2009; Rico et al., 2011). With the present research, we intend to contribute to filling this gap in the literature by clarifying how expressing emotions and being affectively committed to the team influence team innovation.

Although emotions are an intra-psychic phenomenon (Frijda, 1988), they can be expressed and communicated through verbal and non-verbal behaviors (Kennedy-Moore and Watson, 2001). Emotional expression provides important informational resources as it informs about attitudes and intentions (Hareli and Rafaeli, 2008). This information will influence the behaviors, feelings, and thoughts of those who observe, affecting the quality of interactions (Reeve, 2015; van Kleef, 2016). In this context, the construct of ECC can shed some light on the study of the impact of emotional expression within teams on team effectiveness.

ECC was first mentioned in the work of Dutton and Heaphy (2003) in the context of the theory of high-quality connections, as one of the basic characteristics of high-quality relationships. At the team level, it refers to the degree to which team members express emotions, whether positive or negative, in a constructive way (Dutton and Heaphy, 2003; Stephens et al., 2013). It is not just about the amount of emotions expressed, but also relates to the diversity of those emotions (Dutton and Heaphy, 2003), and the ability of the relationship to withstand and evolve with that sharing (Stephens et al., 2013).

High-quality relationships have been shown to contribute to perceived psychological safety and learning behaviors (Härtel et al., 2009), leading to the emergence of more creative and innovative behaviors by team members (Schermuly et al., 2013). Conversely, low-quality relationships can become physically and emotionally stressful, harming individuals and teams (Williams and Dutton, 1999).

Emotional expression is a natural and adaptive process, capable of promoting closer relationships (Reeve, 2015). Thus a team environment characterized by high levels of ECC, in which team members feel that both positive and negative emotions can be expressed without leading to negative consequences, will create the conditions necessary to promote the implementation of innovative ideas (Carmeli, 2009). Indeed, previous studies reveal the positive influence of emotional expression on the emergence of creative ideas (Berg et al., 2017) on team learning and performance (Brueller and Carmeli, 2011) and also on the capacity for knowledge creation (Stephens and Carmeli, 2016). When emotions are shared in a genuine and constructive way, team members tend to be more receptive to divergent opinions and may be more available to jointly find new problem-solving strategies and to be innovative. The positive influence of emotional expression on team innovation may be explained via the broaden-and-build process (Rhee, 2007): expressing emotions stimulates team members to engage in favorable interactions and to share and discuss information, which are essential in finding new solutions and implementing new ideas and processes. Accordingly, we formulate the following hypothesis:

Hypothesis 1 (H1): Emotional carrying capacity is positively related to group innovation.

The opportunity for self-expression and the perception of organizational support (Meyer and Allen, 1991) have been identified as antecedents of affective commitment (Klein et al., 2012), that is, the psychological bond that members feel toward their team (Neininger et al., 2010; Klein et al., 2012). Affective commitment is one of the components of organizational commitment and has been identified as an important predictor of employees’ attitudes and behaviors (Allen and Meyer, 1996). In the present study, we will focus on team affective commitment, which is reflected in a strong emotional connection, high involvement and identification with the team’s goals and values, and a desire to continue belonging to the group (Allen and Meyer, 1990; Darvish and Rezaei, 2011).

Previous research revealed that the ability of the leader to express emotions, values and motives in a transparent and authentic way contributes positively to team commitment (Mazutis and Slawinski, 2008; Darvish and Rezaei, 2011; Paolucci et al., 2018), clearly highlighting the importance of emotional transparency for commitment. In the emotions as social information (EASI) model, van Kleef (2016) identifies affective reactions as one of the two mechanisms that explains the effects of emotional expression on behaviors (the other is inferential processes). As affective commitment is a positive affective emergent state, we may consider that a context in which members sincerely and openly express their emotions contributes to reinforcing their affective attachment to the group. Thus, the following research hypothesis is established:

Hypothesis 2 (H2): Emotional carrying capacity is positively related to affective commitment.

Unlike ECC, commitment is a concept that has been widely explored in team research. However, the relationship between team affective commitment and innovation is not consensual. Indeed, previous empirical research has produced mixed results regarding this relationship (e.g., Zheng et al., 2010; Bastos et al., 2019).

On the one hand, affectively engaged employees tend to experience positive emotions and higher levels of intrinsic motivation (Battistelli et al., 2013), which in turn promote access to innovative ideas and solutions, stimulating individual creativity (Odoardi et al., 2019). Also in this sense, the fact that high affective commitment is associated with organizational citizenship behaviors and loyalty to the organization and team (Meyer and Allen, 1991), as mentioned above, will make it more likely that individuals who are highly committed to the team and the organization will be seen by their supervisors as trustworthy. As a result, their access to the resources needed to put creative ideas into practice may be facilitated (Odoardi et al., 2019). On the other hand, high levels of commitment may result in excessive trust and respect for traditional organizational policies, procedures, and practices, which may diminish the flexible thinking needed to explore and implement creative ideas and solutions (Odoardi et al., 2019; Dimas et al., 2021).

With the present research, we intend to contribute to clarifying the nature of the relationship between team affective commitment and team innovation. Specifically, grounded on social exchange theory, we expect that stronger affective connections of team members toward the group will generate the desire to reciprocate by exchanging ideas and experimenting with procedures and solutions (Cropanzano et al., 2017). Accordingly, we formulate the following hypothesis:

Hypothesis 3 (H3): Affective commitment is positively related to group innovation.

Beyond the direct relationship that we predict between emotional expression and team innovation, we also predict an indirect relationship via team affective commitment. Indeed, building on the EASI model (van Kleef, 2016), and extending it from the interpersonal to the team level, we expect that the affective connection of team members toward the group will act as an affective mechanism through which emotional expression will positively influence team innovation. That is, we consider that a context in which members share their emotions in a genuine way promotes greater bonding among the group, generating, in turn, a greater propensity for the introduction of innovative ideas. Thus, we present the following empirical hypothesis:

Hypothesis 4 (H4): Affective commitment plays a mediating role in the relationship between emotional carrying capacity and group innovation.

**FIGURE 1:**
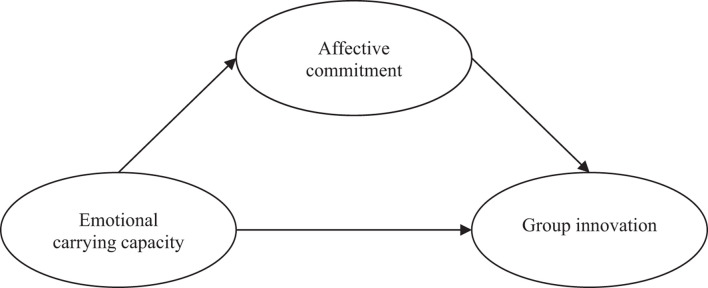
Hypothesized model.

To summarize, this study aims to analyze the relationships between ECC, affective commitment and group innovation. More specifically, the main objective is to analyze the relationship between ECC and group innovation, considering the mediating role of affective commitment. Thus, the hypothetical model represented in Figure 1 will be tested.

## Materials and Methods

### Data Collection Procedures and Sample

The organizations were selected by convenience (Robson and McCartan, 2016), using the personal and professional contacts network of the research team. To collect the data, key stakeholders in each organization (CEOs or human resources managers) were contacted to explain the purpose and requirements of the study. When organizations agreed to participate, the selection of the teams for the survey was based on the following criteria (Cohen and Bailey, 1997; Lourenço et al., 2014): teams must be composed of at least three members, excluding the leader; should be perceived by themselves and other as a team; have to regularly interact interdependently to accomplish a common goal; and must have a formal supervisor who is responsible for the actions of the team. Likewise, receiving responses from at least half of the team members was an inclusion criterion, to ensure the representativeness of the team members’ responses.

Data collection was then carried out through surveys filled in face-to-face or online. In each team, two sources of information were obtained: team members were surveyed about ECC and affective commitment, while team leaders were surveyed regarding team innovation. The use of surveys to collect information proved to be an appropriate method for the objectives of the investigation, since it allows a large amount of data to be collected in a short period of time, with low costs (Robson and McCartan, 2016). The use of the online version also allowed coverage of geographically dispersed individuals. In both cases, the processes of data management, collection and processing followed the ethical assumptions of research in psychology, with the provision of informed consent, and ensuring the anonymity and confidentiality of data and participants (Academy of Human Resource Development Standing Committee on Ethics and Integrity, 1999; Ordem dos Psicólogos Portugueses, 2016).

Initially, the sample had 169 teams. However, 31 teams were excluded due to a response rate of team members below 50%, the lack of a response from the leader or for the presence of questionnaires where at least 10% of the responses were missing (Bennett, 2001; Bryman and Cramer, 2004). Thus, the sample on which the study was focused was reduced to 138 work teams, consisting of 625 members and their leaders, from 89 organizations.

The most represented organizations are considered large, with more than 250 employees (31.9%), followed by small organizations with no more than 10 employees (28.9%). Regarding the sector of activity, the majority (66.4%) are from the commerce and services sector. Likewise, the work teams also differ in their area of activity, with the most common being the services area (35.8%). As for the seniority of the teams, it ranges between less than 1 and 46 years approximately (*M* = 8.03; *SD* = 8.92). The size of the team varies between three and 22 members, with the average being approximately six (*SD* = 3.78).

The 625 members of the participating teams were between 17 and 67 years old (*M* = 36.27; *SD* = 11.46) and were mostly female (60.7%). Regarding academic qualifications, undergraduate degrees were the most represented (42.9%). Seniority in the organization ranged from less than 1 to 50 years (*M* = 9.78; *SD* = 10.04), and seniority in the team ranged from less than 1 to 43 years, approximately (*M* = 5.13; *SD* = 6.13). The majority (55.1%) reported having had training in teamwork.

The 138 leaders were between 18 and 67 years old (*M* = 36.27; *SD* = 11.46), mostly male (57.5%), and most reported having a college degree (57.0%). On average, they had been with the organization for 13.86 years (*SD* = 10.58), ranging from less than 1 to 47 years, and had led the team in question for an average of 5.63 years (*SD* = 6.42), ranging from less than 1 to 47 years.

### Measures

All scales were used in their Portuguese versions.

#### Emotional Carrying Capacity

The scale used to assess ECC was based on Stephens et al. (2013) proposal for assessing this capacity in top management teams, which in turn had been adapted from Carmeli’s (2009) High-Quality Relationships scale.

The team members were asked to rate the three items using a 5-point Likert-type scale, where 1 was “Strongly Disagree” and 5 was “Strongly Agree.” A sample item is “Team members have no problem expressing their feelings toward each other.”

In the adaptation to the Portuguese version, the psychometric qualities of the scale were assessed by Brito (2020), obtaining a Cronbach’s alpha value of 0.80, whereas in the study by Stephens et al. (2013), this value was 0.71.

#### Affective Commitment

In order to measure this construct, a scale was used composed of four items from Allen and Meyer (1990) affective commitment scale, adapted by Han and Harms (2010). The team members were asked to rate each statement using a 5-point Likert-type scale, ranging from 1, “Strongly Disagree,” to 5, “Strongly Agree.” A sample item is “Team members have a strong sense of belonging to the team.”

In the adaptation to the Portuguese version, the psychometric qualities of the scale were assessed by Bastos et al. (2019), obtaining a Cronbach’s alpha value of 0.85, whereas in the second study by Han and Harms (2010), this value was 0.92.

#### Group Innovation

A three-item scale was used based on Batarseh et al. (2017). A sample item is “The team is highly innovative.” Team leaders were asked to rate each item using a 7-point Likert-type scale, where 1 corresponded to “Strongly Disagree” and 7 to “Strongly Agree.”

Similarly to the other instruments, the scale was translated into Portuguese and validated in a previous study (Bastos et al., 2019), obtaining a Cronbach’s alpha value of 0.82, whereas in the study by Batarseh et al. (2017), this value was 0.89.

### Control Variables

Previous studies show that team size has an influence on group processes and outcomes (Wheelan, 2009). However, the impact of the number of team members on innovation processes is not consensual. West and Altink (1996a) report that the larger the team, the less likely effective and successful innovation attempts are. Hülsheger et al. (2009), on the other hand, report a positive and significant relationship between team size and group innovation.

Similarly, team seniority has also been reported as potentially influencing group processes, particularly commitment (Ilgen et al., 2005). Wheelan (2009) mentions that commitment tends to increase when the group goes beyond the developmental stage characterized by internal conflicts.

Both variables were included as control variables, and the information regarding them was collected from the leaders.

### Data Analysis and Previous Procedures

After the invalid teams were removed, the number of missing values in the members’ sample was quite small (only three) and no missing values were identified in the leaders’ sample. Little’s MCAR test was used to evaluate the distribution pattern of the missing values. Since the test pointed to a random distribution of missing values (*p* ≥ 0.05), these values were replaced by the mean of the respective item (Hair et al., 2019).

Then, to justify the aggregation of data concerning ECC and affective commitment, the values of the James index, or r_wg_ (James et al., 1984) and the intraclass correlation coefficients ICC (1) and ICC (2) were calculated for these scales. For the r_wg_, the mean values obtained were 0.81 for ECC and 0.87 for affective commitment. Since both are above 0.70, the reference value, it can be considered that there is agreement among members (Lebreton et al., 2003). As for the intraclass correlation coefficients, the ICC (1) values found for ECC and affective commitment were 0.22 and 0.28, respectively, so they are congruent with what is suggested (e.g., above 0.10 according to Bliese, 1998). In turn, the ICC (2) values for the same variables were 0.56 and 0.64, respectively, also in line with what is suggested (e.g., above 0.50 according to Klein and Kozlowski, 2000). Thus, data aggregation was justified, and this procedure was performed by calculating the mean scores of the members of each group for each item.

Subsequently, an analysis of the correlations between the variables under study and the control variables was performed. To perform these procedures and to calculate the descriptive statistics used to describe the sample, the IBM SPSS Statistics software (version 25) was used.

Structural equation modeling (SEM) and AMOS software were then used to evaluate the hypothetical model under analysis (see Figure 1). Since the distributions of the variables were close to normality, the maximum likelihood (ML) estimation method was chosen. Following the recommendations of authors such as Kline (2016), the two-step modeling procedure was used. In the first stage, the use of confirmatory factor analysis (CFA) allowed the items’ relationships with the latent variables to be assessed, thus testing the measurement model (Kline, 2016). In the second stage, SEM was performed with the aim of assessing the relationships between the variables under study and testing the research hypotheses.

In order to ensure that the assumptions of the SEM were met, the uni- and multivariate normality of the variables was assessed by the skewness (sk) and kurtosis (ku) coefficients. To evaluate the overall adjustment quality of the model, the χ^2^-test of the adjustment was taken into account, as well as the following indexes: the chi-square/degrees of freedom (χ^2^/gl), the result of which should be less than 2 to be considered a good adjustment (Kline, 2016); the root mean square error of approximation (RMSEA), the criterion of which is a value less than 0.05 to be considered a very good adjustment, and a non-significant *p-*value (*p* > 0.05) (Kline, 2016); and the Tucker-Lewis index (TLI), the comparative fit index (CFI), and the incremental fit index (IFI), which must present values greater than 0.90 to be considered a good adjustment (Kline, 2016). The quality of the local adjustment was assessed by the factorial loadings and the individual item reliability.

Finally, the bootstrapping method was used to test the statistical significance of the indirect effects contained in the structural model.

## Results

### Psychometric Qualities of the Instruments

Preliminary analysis revealed that no variable showed asymmetry (sk < 3) or kurtosis (ku < 10) coefficients indicative of severe violations of the normal distribution (Kline, 2016). Given the values of the indicators listed above, the measurement model revealed a good fit to the data [χ^2^(32) = 31.249, *p* = 0.504; χ^2^/gl = 0.98; IFI = 1.001; TLI = 1.001; CFI = 1.000; RMSEA = 0.000, IC 90% 0.000–0.061, *p* = 0.878].

Additionally, this three-factor model was compared with a two-factor model (where one factor comprised all the items answered by the members and the other factor the items answered by the leaders), as well as with a single-factor model (where all the items loaded on one latent factor) to provide evidence to overcome concerns of potential common method variance bias (Chang et al., 2010). The three-factor model shows a superior fit, with the difference between this model and the others being statistically significant: Δχ^2^(34) = 127.058, *p* < 0.001, and Δχ^2^(35) = 328.141, *p* < 0.001 concerning the two-factor and the single-factor models, respectively.

The measures used show high internal consistency, as shown in Table 1, through composite reliability values (Kline, 2016) and Cronbach’s alpha (Nunnally, 1978) greater than 0.7 across all constructs.

**TABLE 1:** Standardized factorial loadings and individual reliability of items, composite reliabilities, Cronbach’s alpha, and average variance extracted of measures.

**Variables**	**Item**	**λ**	**λ ^2^**	**CR**	**α**	**AVE**
Emotional carrying capacity	1	0.82	0.67	0.85	0.85	0.66
	2	0.79	0.62			
	3	0.83	0.68			
Affective commitment	1	0.80	0.63	0.92	0.92	0.75
	2	0.86	0.73			
	3	0.93	0.87			
	4	0.88	0.78			
Group innovation	1	0.86	0.74	0.89	0.89	0.72
	2	0.82	0.68			
	3	0.86	0.75			

*λ, factorial weight; λ^2^, standardized factorial weight; CR, composite reliability; α, Cronbach’s alpha; AVE, average variance extracted.*

Once the reliability of the scales was ensured, it was necessary to assess the construct validity, considering convergent, discriminant and factor validity. Convergent validity, assessed by the average variance extracted (AVE), proved to be adequate, presenting values greater than 0.5 in all factors (Fornell and Larcker, 1981; Hair et al., 2019; see Table 1). In turn, the discriminant validity of the factors was assessed by comparing the AVE with the squares of the correlation between factors, as proposed by Fornell and Larcker (1981). Since AVE_ECC_ = 0.66 and AVE_AC_ = 0.75 are higher than r^2^_ECC.AC_ = 0.43, it can be stated that the two factors have discriminant validity. Similarly, the discriminant validity of the factors “Affective Commitment and Innovation” and “Emotional Carrying Capacity and Innovation” was demonstrated, with the squared correlations, r^2^_A__C.INOV_ = 0.12 and r^2^_E__CC.INOV_ = 0.08, respectively, being considerably lower than the AVE values of each of the factors. Finally, standardized factor loadings greater than 0.5, and individual item reliability greater than 0.25, are indicators of factor validity (Hair et al., 2019).

### Hypothesis Testing

Table 2 presents descriptive statistics and correlations between the variables under study. Since team seniority is significantly related to affective commitment, and team size to affective commitment and innovation, it was justified to include them as control variables in the structural model. Therefore, it was necessary to assess the adjustment of the structural model, and this also revealed a good fit [χ^2^(47) = 51.840, *p* = 0.291; χ^2^/gl = 1.103; IFI = 0.995; TLI = 0.993; CFI = 0.995; RMSEA = 0.027, IC 90% 0.000–0.064, *p* = 0.812].

**TABLE 2:** Descriptive statistics and correlation matrix.

**Variables**	**M**	**SD**	**1**	**2**	**3**	**4**	**5**	**6**
Emotional carrying capacity	3.60	0.52	−					
Affective commitment	3.84	0.53	0.60**	−				
Group innovation	5.10	1.00	0.26**	0.34**	−			
Team seniority	8.03	8.92	−0.25**	−0.18*	0.02	–		
Team size	6.21	3.78	−0.18*	−0.17*	−0.23**	0.19*	–	

N = 138. *correlation is significant at the p < 0.05 level (2-tailed); ** correlation is significant at the p < 0.01 level (2-tailed).

**FIGURE 2:**
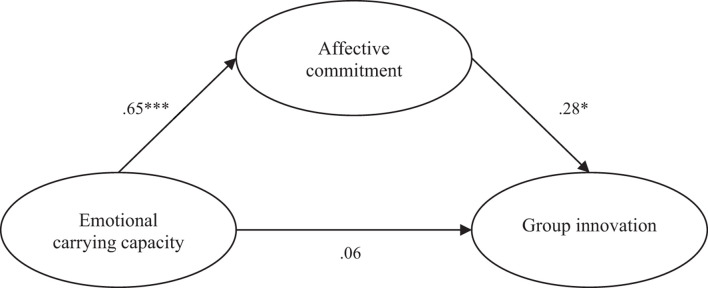
The SEM analysis conducted to examine pathways among emotional carrying capacity, affective commitment, and group innovation. ^∗^*p* < 0.05 (2-tailed); ^∗∗∗^*p* < 0.001 (2-tailed).

Observing the direct relationships of the structural model (cf. Table 3), a statistically significant relationship is found between ECC and affective commitment, which supports Hypothesis 2, as well as a significant relationship between affective commitment and group innovation, supporting Hypothesis 3. On the other hand, the relationship between ECC and group innovation was found not to be statistically significant, thus not supporting Hypothesis 1. Results are summarized in Figure 2.

**TABLE 3:** Summary of the structural model paths.

**Paths**	**Hypotheses**	**Standardized estimates**	***t*-value**
ECC → Group innovation	H1	0.06	0.49
ECC → Affective commitment	H2	0.65	6.47***
Affective commitment → Group innovation	H3	0.28	2.17*
Team seniority → Affective commitment		–0.01	–0.10
Team size → Affective commitment		–0.04	–0.60
Team size → Group innovation		–0.19	−2.18*

N = 138; *significant at the p < 0.05 level (2-tailed); ***significant at the p < 0.001 level (2-tailed); ECC, Emotional carrying capacity.

To assess the mediating effect, a resampling bootstrapping procedure and 2,000 samples were used with a 95% confidence interval for two-sided tests. Thus, the estimate of the indirect effect of ECC on group innovation through commitment is framed by a 95% confidence interval, with bounds [0.002; 0.397], presenting a significance value lower than 0.05, supporting Hypothesis 4.

## Discussion

The results supported the positive relationship between ECC and affective commitment, supporting Hypothesis 2 (H2).

The results are in line with those of Meyer and Allen’s (1991) research, which suggest that the opportunity for personal expression and the perception of organizational support, which are characteristics of teams with high ECC, are related to affective commitment. Accordingly, Darvish and Rezaei’s (2011) results also showed a positive relationship between leaders’ ability to be authentic in their communication (i.e., leader transparency) and team affective commitment. This indicates that transparency in the expression of emotions by team members may also be a promoter of commitment. In fact, considering that the leader’s relational transparency implies the ability to communicate with team members in a genuine way, particularly their emotions, it is possible to establish a parallel between the leader’s ability and the concept of expression of emotions, presented above (Darvish and Rezaei, 2011). Indeed, if team members feel more comfortable and safer in sharing their emotions, there will be a greater tendency to build a strong emotional bond, and a high involvement and identification with the team’s goals and values (Allen and Meyer, 1990; Darvish and Rezaei, 2011).

Regarding the relationship between ECC and group innovation (H1), contrary to our expectations, the results did not support this relationship. Additionally, it should be noted that, although a direct relationship between these variables was not identified, an indirect relationship through affective commitment was found, providing empirical support for Hypothesis 4 (H4). That is, ECC promotes team innovation because it generates commitment, which in turn increases innovation. The results are in line with those of Stephens et al. (2013), which suggest that sharing positive emotions seems to contribute to a greater adaptive capacity of the team.

The results reinforce the notion that constructs with a positive affective component tend to promote positive group attitudes and processes (e.g., Fredrickson and Joiner, 2002; Ashkanasy and Dorris, 2017; Blázquez-Puerta and Bermúdez-González, 2019). Indeed ECC is characterized by its affective component (Stephens et al., 2013), and seems to be related to other positive constructs, such as psychological security in relationships (Berg et al., 2017), team learning and performance (Brueller and Carmeli, 2011), and, as our results showed, also to affective commitment.

Finally, the results show that affective commitment has a significant positive effect on group innovation, which supports the third hypothesis of the study (H3). Although the relationship between these variables has already been studied, the positive and significant relationship found is in line with the results of previous studies (Jafri, 2010; Zheng et al., 2010), suggesting that team members, when are affectively committed, strive to propose innovative suggestions to contribute to group results (Jafri, 2010). This relationship may be the result of several factors. For instance, the tendency for affectively engaged employees to experience higher levels of motivation (Battistelli et al., 2013), more organizational citizenship behaviors and loyalty to the team (Meyer and Allen, 1991) promotes their desire to contribute to the team by presenting suggestions and implementing them (Odoardi et al., 2019).

### Theoretical Contributions and Practical Implications

This study contributes to the literature in different ways. To the best of our knowledge this is the first study that considers the direct and indirect influence of team members’ capacity to express emotions on team innovation. By highlighting the influence that team members’ ECC has on their ability to innovate and, specifically, by presenting affective commitment as a mechanism involved in this relationship, our study contributes to team innovation literature, supporting the fundamental role that affective dimensions have on this important team outcome. Likewise, our study contributes to the growing, but still underdeveloped, literature on emotional expression in groups (van Kleef, 2016) by providing support for the importance that the capacity of expressing both positive and negative emotions appropriately has on team states (i.e., affective commitment) and outcomes (i.e., team innovation).

Second, our study reveals that when team members are able to express themselves fully, they feel more connected to the team.

Finally, our study contributes to clarifying those mechanisms through which ECC is promoted have an impact on team members’ ability to innovate, presenting affective commitment as a key mediator variable in this relationship.

From a practical point of view, focusing on the antecedents of an outcome (i.e., team innovation) that is crucial in the complex and dynamic environment in which modern organizations are embedded, our study provides guidelines about how to promote innovation in work teams. Specifically, the results highlight the importance of creating a space where members are able to fully express their emotions themselves, which leads to committing to and identifying with the team’s goals and values, enabling them to speak up, suggest new ideas and take risks (Stephens et al., 2013). This healthy bond that links team members to the group will increase their desire to exert efforts on behalf of the group, which will eventually lead to innovation.

Given this relationship, it is important for leaders to promote healthy bonds between team members, characterized by friendly and welcoming interpersonal styles, mutual respect, and awareness of the needs and concerns of others (Richardson and West, 2009). That can be done by investing in inter-personal relationships and by adopting an opening and welcoming conduct when receiving suggestions, new ideas, different opinions or when addressing polemic issues. Managers can stimulate the sharing of experiences in order to regulate the team’s emotional state during initial interactions (Yang and Kelly, 2016).

The development of such relationships will contribute not only to an increase in affective commitment and innovation, but also to more helping behaviors and higher levels of job satisfaction (Ashkanasy and Humphrey, 2011), which positively contribute to the well-being of employees (Ashkanasy and Dorris, 2017).

### Limitations and Future Directions

Although this study contributes to a better understanding of the constructs analyzed, it presents some limitations. First, the convenience sampling used, the fact that the sample consists only of Portuguese organizations, and that more than 66% of them operate in the trade and services sector implies caution in generalizing the results (Robson and McCartan, 2016).

Second, the cross-sectional design is an obstacle to the empirical inference of causality, so it would be appropriate to conduct a longitudinal study. Third, regarding the measurement instruments, the use of self-report measures constitutes a limitation, since the information collected is related to the evaluation that individuals make of their own group and may reflect the effect of social desirability (Robson and McCartan, 2016). However, the fact that the responses were evaluated at the group level mitigates this limitation, since several people assessed the same phenomenon (Podsakoff et al., 2012). Additionally, the exclusive use of self-report measures may have contributed to the occurrence of the common method bias, namely considering ECC and affective commitment, which were obtained from the same source (i.e., team members). However, it is important to highlight that different proactive procedures were implemented in order to minimize threats of common method bias: respondents’ anonymity was ensured, which reduces evaluation apprehension; variables were evaluated through previously validated scales that were constituted by concise, simple, and specific items (i.e., items are not ambiguous and show lack of overlap for the different constructs); although obtained in the same survey, the scales were separated and specific instructions were provided for each scale (Conway and Lance, 2010; Podsakoff et al., 2012). Moreover, it is important to highlight that one of the most important procedures to control for common-method bias, which is collecting data from multiple sources, was implemented in this study (Chang et al., 2010).

Finally, the sample size may also constitute a limitation, given the statistical analysis that was performed. Although the sample does have an average size to perform SEM, between 100 and 200 cases, the model is not parsimonious, presenting a ratio of 4.45, that is, very close to the cut-off of 5:1 (Mulaik et al., 1989; Kline, 2016). Thus, the suggestion is to replicate this study with a larger sample in order to get a more acceptable value of the ratio between the number of cases and the number of estimated parameters.

## Data Availability Statement

The raw data supporting the conclusions of this article will be made available by the authors, without undue reservation.

## Ethics Statement

The studies involving human participants were reviewed and approved by the Ethics Committee of the Faculty of Psychology and Education Sciences of the University of Coimbra. The patients/participants provided their written informed consent to participate in this study.

## Author Contributions

RD: data collection, data analysis, and writing the manuscript. ID: design of the study, data collection, writing the manuscript, data analysis, review, and editing. PL and TR: design of the study, data collection, review, and editing. MA: design of the study, review and editing. All authors contributed to the article and approved the submitted version.

## Conflict of Interest

The authors declare that the research was conducted in the absence of any commercial or financial relationships that could be construed as a potential conflict of interest.

## Publisher’s Note

All claims expressed in this article are solely those of the authors and do not necessarily represent those of their affiliated organizations, or those of the publisher, the editors and the reviewers. Any product that may be evaluated in this article, or claim that may be made by its manufacturer, is not guaranteed or endorsed by the publisher.

## Footnotes

1 In the present paper, and following other authors (e.g., Mathieu et al., 2017), the terms group and team will used interchangeably.
